# Extraction of Bioactive Compounds Using Supercritical Carbon Dioxide

**DOI:** 10.3390/molecules24040782

**Published:** 2019-02-21

**Authors:** Antonio Molino, Vincenzo Larocca, Giuseppe Di Sanzo, Maria Martino, Patrizia Casella, Tiziana Marino, Despina Karatza, Dino Musmarra

**Affiliations:** 1 Department of Sustainability—CR Portici, Italian National Agency for New Technologies, Energy and Sustainable Economic Development (ENEA), P. Enrico Fermi, 1, 80055 Portici (NA), Italy; patrizia.casella@enea.it; 2 Department of Sustainability—CR Trisaia, Italian National Agency for New Technologies, Energy and Sustainable Economic Development (ENEA), SS Jonica 106, km 419+500, 75026 Rotondella (MT), Italy; vincenzo.larocca@enea.it (V.L.); giuseppe.disanzo@enea.it (G.D.S.); maria.martino@enea.it (M.M.); 3 Institute on Membrane Technology, National Research Council (ITM-CNR) Via Pietro Bucci, Cubo 17C, 870 36 Rende (CS), Italy; t.marino@itm.cnr.it; 4 Department of Engineering, University of Campania “L.Vanvitelli”, Real Casa dell’Annunziata, Via Roma 29, 81031 Aversa (CE), Italy; karatza@irc.cnr.it (D.K.); dino.musmarra@unicampania.it (D.M.)

**Keywords:** Microalgae, *Dunaliella salina*, β-carotene, fatty acids, supercritical-CO_2_ fluid extraction, food additives

## Abstract

Microalgae *Dunaliella salina* contains useful molecules such as β-carotene and fatty acids (FAs), which are considered high value-added compounds. To extract these molecules, supercritical carbon dioxide was used at different operative conditions. The effects of mechanical pre-treatment (grinding speed at 0–600 rpm; pre-treatment time of 2.5–7.5 min) and operating parameters for extraction, such as biomass loading (2.45 and 7.53 g), pressure (100–550 bars), temperature (50–75 °C) and CO_2_ flow rate (7.24 and 14.48 g/min) by varying the extraction times (30–110 min) were evaluated. Results showed that the maximum cumulative recovery (25.48%) of β-carotene was achieved at 400 bars and 65 °C with a CO_2_ flow rate of 14.48 g/min, while the highest purity for stage (55.40%) was attained at 550 bars and 65 °C with a CO_2_ flow rate of 14.48 g/min. The maximum recovery of FAs, equal to 8.47 mg/g, was achieved at 550 bars and 75 °C with a CO_2_ flow rate of 14.48 g/min. Moreover, the lowest biomass loading (2.45 g) and the first extraction cycle (30 min) allowed the maximum extraction of β-carotene and FAs.

## 1. Introduction

In the last decade, the international market of natural products for human health has noticeably increased; in fact, the sales volumes are higher than that of similar synthetic products because end users seem to pay more attention to quality than before. β-carotene represents one of the most important players in the market of natural products, with about 350 million euros of revenue as of 2015, and is estimated to increase by 2023 to about 450 million euros [1,2,3]. Nowadays, the greatest portion of the β-carotene market, accounting for about 90%, is the food additives sector, while 7% is requested as feed additives and only 3% is used in the cosmetic field. The β-carotene market is covered mainly from synthetic forms (over 97%) and only 2–3% is derived from biological sources [4,5], that is, mainly plants, vegetables and fruits (apricots, grapes, mangoes, persimmon, watermelon, carrots, sweet potatoes, tomato and spinach) [6], but it can also be derived from bacteria, mushrooms and microalgae [7,8,9]. Natural β-carotene, produced for the international market, is extracted from *Blakeslea trispora* fungi as well as *Dunaliella salina* microalgae, which have been approved by the Food and Drug Administration (FDA) and Joint FAO/WHO Expert Committee on Food Additives (JECFA). The increasing awareness of the major uses of natural products has gain further evidence of the importance to substitute synthetic compounds with natural analogues, as in the case of carotenes produced from *D. salina* microalgae. β-carotene is one of the 600 carotenoids available in the natural ecosystem [10], having two cyclic rings composed of six carbon atoms at its extremity [11] as its main structural characteristic. The main properties of β-carotene are related to its antioxidant effects. Moreover, it is a precursor of vitamin-A that promises several human health benefits for visual embryonic development, epithelial cells and the immune system [12,13]. Thanks to these properties, β-carotene is used in several industrial markets including food, cosmetic and nutraceuticals. In the food industries, it is used as a coloring agent in food (margarines) and drinks (juices) [14], while in the cosmetic field it is used as a protective agent against UV rays. In the nutraceutical market it is used as vitamin supplement [15]. Lipids are other interesting compounds that can be extracted from several microalgal species. They are composed mainly of triglycerides that can be easily converted in fatty acids (FAs). It is possible to distinguish saturated fatty acids (SFAs), monounsaturated fatty acids (MUFAs) and polyunsaturated fatty acid (PUFAs). The FAs offer several major health benefits such as reducing triglycerides in the blood serum, decreasing blood pressure and decreasing the incidence of tumors [16].

These molecules can be extracted from *D. salina* microalgae, which belongs to the class Chlorophyta (green algae) and is characterized by a pseudo-circular form with two peduncles used for moving, with a cell length in the range of 16–24 µm [17,18]. It lives in marine and salty environments and is able to tolerate salinity concentrations up to 4 M NaCl [19]. *D. salina* is able to accumulate an average amount of β-carotene equal to 2–3% *w/w* on dry basis, but under high stress conditions can accumulate up to 10% *w/w* and 44% *w/w* on a dry basis of β-carotene and total lipids, respectively [20,21]. Furthermore, extracts from *D. salina* contain other carotenoids such as lutein and zeaxanthin, which have several antioxidant effects [22]. In terms of β-carotene composition, *D. salina* contains both trans β-carotene isomers (50%) and 9-cis isomers (50%), which are important for conversion to retinoic acid, while synthetic β-carotene contains only its trans form [23]. Synthetic β-carotene was produced for the first time by Roche (1954) and BASF (1960) and is currently produced starting from β-ionone, which is derived from acetone or butadiene [24]. *D. salina* is mainly cultivated using open ponds as raceway pond systems. There are around 10 industrial producers of *D. salina* with an annual production of about 1200 tons of dry biomass [25]. After the cultivation step, the microalgae are separated from the aqueous medium by means of centrifugation and flocculation techniques, and are dried before their use in the extraction process [26]. The processes of harvest, concentration and drying are the most important ones for two reasons. The first is linked to the low algal concentration that is usually up to 0.1 g/L; therefore, the concentration process becomes more laborious and expensive. The second reason is related to the absence of a rigid wall in the structure of *D. salina*. During these stages, it is important to avoid the break of the cellular wall and the loss of organic compounds. β-carotene and FAs are extracted from *D. salina* on an industrial scale by using conventional extraction technologies, primarily based on solid-liquid extraction. Otherwise, literature data show that β-carotene can be extracted from *D. salina* by innovative techniques such as pressurized liquid extraction (PLE), ultrasonic and microwave assisted extractions (UAE and MAE) [27]. Supercritical Fluid Extraction (SFE) has been in development for around five decades, so has been recognised for its potential for some time, and is especially useful for the extraction of heat sensitive compounds, such as β-carotene, to minimize thermal degradation. In addition, this technique avoids the use of toxic solvents: CO_2_ is a green solvent and is cheap and easy to separate from the extract [28,29]. Furthermore, SFE using CO_2_ does not require separation and purification of the extract from the organic solvent, since CO_2_ is a gas at room temperature and pressure. Despite some authors having already used supercritical extraction technology for the extraction of carotenoids and FAs from vegetables and several species of microalgae, such as *Haematococcus pluvialis* and *Chlorella. vulgaris* [30,31,32,33,34,35]. This work reports the effects of mechanical pre-treatment at 0–600 rpm for 2.5–7.5 min and operating parameters including an extraction time of 30–110 min, biomass loading at 2.45 and 7.53 g, temperature at 50–75 °C, pressure at 100–550 bars and CO_2_ flow rates of 7.24 and 14.48 g/min, on the extraction of β-carotene and FAs from *D. salina* using SFE-CO_2_ technology. Moreover, the performance of SFE-CO_2_ on FA classes, which are distinguished as SFAs, MUFAs and PUFAs was also investigated. For the first time, recovery and purity of β-carotene for each extraction cycle were analyzed. This method could be implemented in food industries for direct supplementation of food product with different concentrations of β-carotene.

## 2. Results and Discussion

The extraction yield, expressed as mg of extract per g dry weight of biomass, were obtained with an extraction time of 110 min. Total extraction yield varied from a minimum of 3.52 mg/g at 100 bars to a maximum of 39.04 mg/g at 400 bars, operating in both cases at 65 °C, with a *D. salina* biomass loading of 2.45 g and a CO_2_ flow rate of 14.48 g/min. The wide variation range seemed to show the critical role of pressure during the extraction process.

### 2.1. Effect of Mechanical Pre-Treatment on β-carotene Recovery

**Figure 1:**
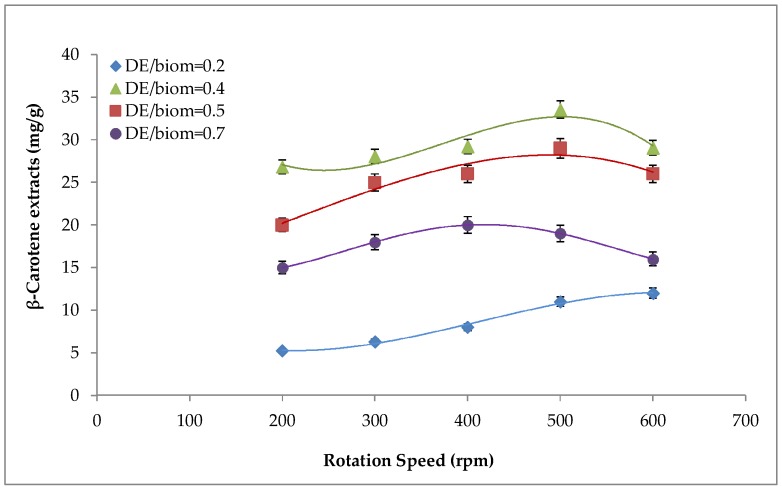
Effect of different rotation speeds on β-carotene recovery.

The first experimental series was carried out to evaluate the effect of the mechanical pre-treatment at different rpm and by varying the ratio between Diatomaceous Earth (DE) and biomass, on the recovery of β-carotene fixed the pretreatment time at 5 min (Figure 1). Figure 1 showed that β-carotene recovery in each operative conditions increase with increasing rpm up to a maximum value depending of the DE/biomass ratio.

**Figure 2:**
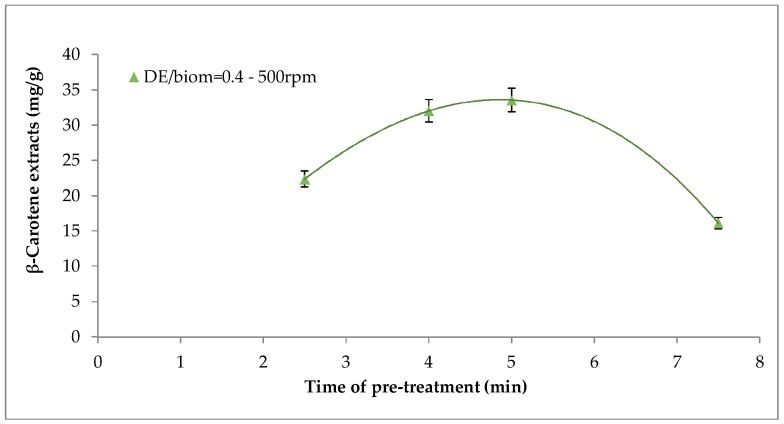
Effect of pre-treatment time at 500 rpm on β-carotene recovery.

Overall, among all tested rpm values, maximum recovery of β-carotene was achieved at 500 rpm. Moreover, the pre-treatment time also played a key role on β-carotene extraction. Among all the tested pre-treatment time, a period of 5 min resulted in the highest β-carotene recovery (98.4%) at 500 rpm. Furthermore, it was possible to observe from Figure 2 that a fixed time of 2.5 min was not sufficient to disrupt the double-walled cell and to break the thylakoids, which are the subcellular structures where β-carotene molecules are located in their ester forms. In contrast, 7 min was enough to enable β-carotene release from the cell body. Therefore, mechanical pre-treatment enhanced the accessibility of the carotenoid bound to the cell organelles during supercritical fluids, thereby increasing the extraction yield.

Our result showed (Figure 2) that pre-treatment for 5 min at 500 rpm with DE/biomass equal to 0.4 constituted the optimum operating conditions for the mechanical pretreatment and, consequently, for all the follow-up Carbon dioxide Supercritical fluid extraction (CO_2_-SFE) tests these conditions were maintained unchanged.

### 2.2. Effect of Pressure on β-carotene and FAs Extraction

**Figure 3:**
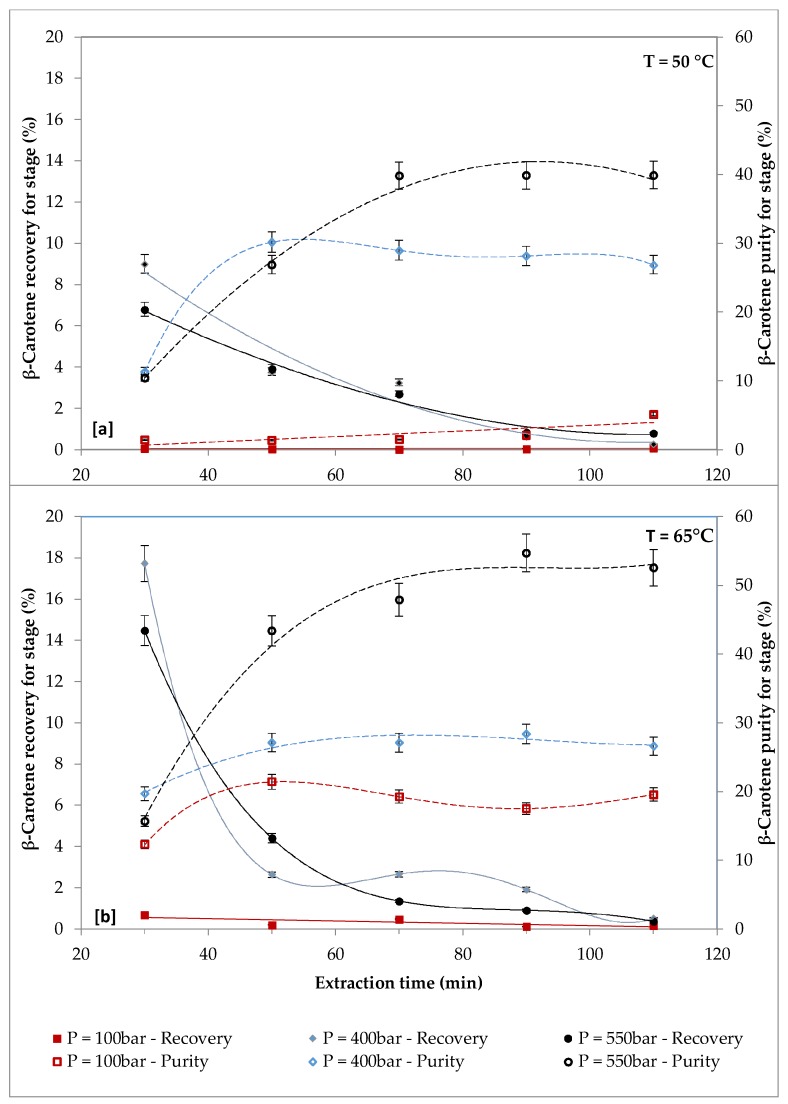

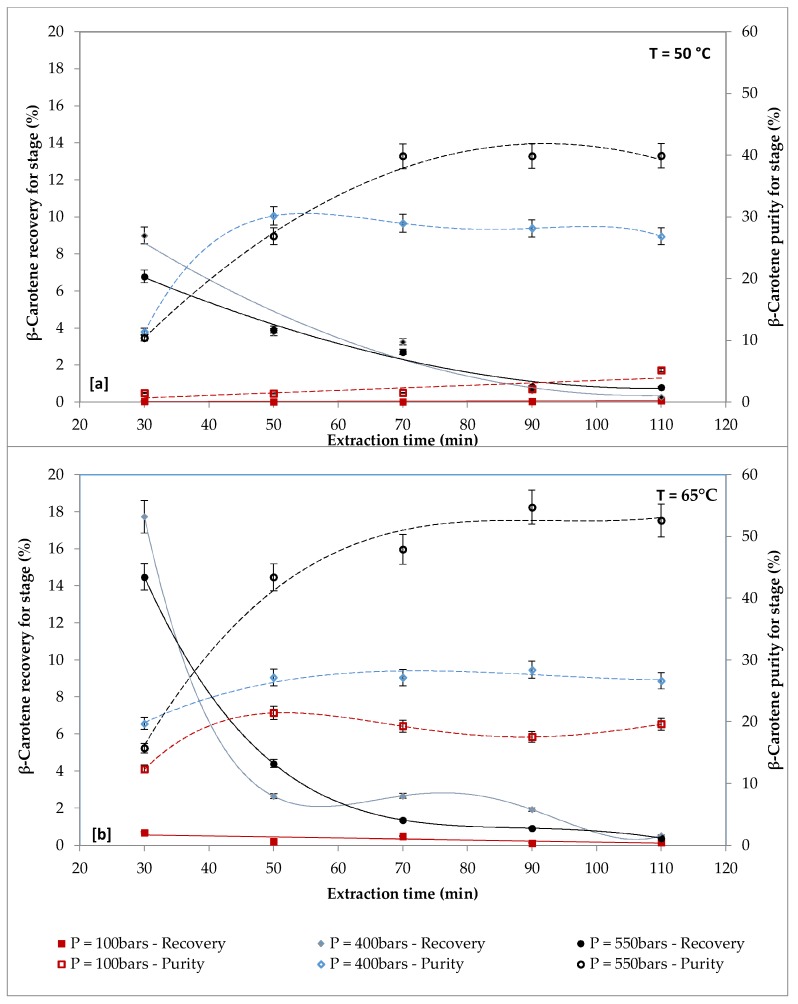
Effect of pressure (100–550 bars) on recovery and purity of β-carotene at different temperatures (biomass loading = 2.45 g, CO_2_ flow rate = 14.48 g/min): [a] Temperature = 50 °C; [b] temperature = 65 °C.

The effect of pressure (100 bars, 400 bars and 550 bars) at two different temperatures (50 °C and 65 °C) on the recovery and purity of β-carotene is shown in Figure 3.

It can be observed that at 50 °C and 65 °C, the highest β-carotene recovery was obtained at the first extraction cycle (30 min) with the intermediate pressure of 400 bars. At a temperature of 50 °C (Figure 3a), the highest recovery of β-carotene was equal to about 9% (first extraction cycle = 30 min, P = 400 bars, CO_2_ flow rate = 14.48 g/min, biomass loading = 2.45 g) and the highest purity of β-carotene was equal to about 40% (third extraction cycle = 70 min, P = 550 bars, CO_2_ flow rate = 14.48 g/min, biomass loading = 2.45 g). At a temperature of 65 °C (Figure 3b), the highest recovery of β-carotene was equal to about 18% (first extraction cycle = 30 min, P = 400 bars, CO_2_ flow rate = 14.48 g/min, biomass loading = 2.45 g) and the highest purity of β-carotene was equal to about 55% (fourth extraction cycle = 90 min, P = 550 bars, CO_2_ flow rate = 14.48 g/min, biomass loading = 2.45 g). Similar findings were observed by other researcher, who investigated the extraction of carotenoids from *Scenedesmus almeriensis* and *Nannochloropsis gaditana*. The authors reported that the highest recovery of carotenoids was observed at 400 bars, even though the maximum pressure tested was 600 bars. These results may be explained by considering the contrasting effect of pressure on the properties of the extracting fluid when pressure increased; the density of CO_2_ increased, enhancing the solvating power of the fluid and the solubility of non-polar molecules. Therefore, a higher extraction yield was obtained, but at the same time, this phenomenon was balanced by a decrease in the diffusion coefficient, which in turn reduced the penetration capacity of the solvent and decreased the extraction yield at higher pressures.

In terms of purity of β-carotene, the highest value was achieved at 550 bars, at the third extraction cycle (70 min) when a temperature of 50 °C was used and at the fourth extraction cycle (90 min) at 65 °C. The β-carotene purity was significantly improved by increasing pressure and extraction time, up to a critical value, above which purity is constant. For both temperatures, the lowest recovery of β-carotene and the lowest purity of β-carotene were found at the pressure of 100 bars. The above-mentioned results indicated that β-carotene exhibited better extraction yield at 400 bars and higher purity at 550 bars.

The characterization of FAs extracted from *D. salina* when varying the operative pressure in the range of 100–550 bars, and at temperatures of 50 °C and 65 °C, is reported in Table 1.

For both the investigated temperature, lipid recovery increases as extraction pressure increases, reaching highest value at 400 bars followed by a decrease. Comparing the extracted amounts with the theoretical contents, it was possible to observe that the maximum recovery of lipids, equal to 7.91 mg/g, was obtained at 65 °C and 400 bars this value corresponds to a recovery of about 23% with respect to the theoretical content. The highest recoveries of SFAs, MUFAs and PUFAs were found at 65 °C and 400 bars, with values of about 4.12, 2.69 and 0.78 mg/g, respectively. In terms of species, palmitic acid and stearic acid were found as SFAs, cis-9-octadecenoic acid was the only MUFA, and linoleic acid-ω-6 was found as the only PUFA.

SFE-CO_2_ is considered a suitable approach for extraction of FAs from microalgae due to its non-polar property. Other research groups [36,37,38,39] reported that SFE-CO_2_ is selective for neutral lipids (non-polar lipids), but did not solubilize polar lipids. Results showed that by using CO_2_-SFE technology and, therefore, a non-polar solvent, only a fraction lower than 21% (7.91mg/g lipids) is recovered from extraction and this could be due to the solubility of triglyceride molecules with respect to carbon dioxide. It is possible to suppose that only triglycerides that have solubility like carbon dioxide are appropriate for this extraction process.

### 2.3. Effect of Biomass Loading on β-Carotene and on FAs Extraction

**Figure 4:**
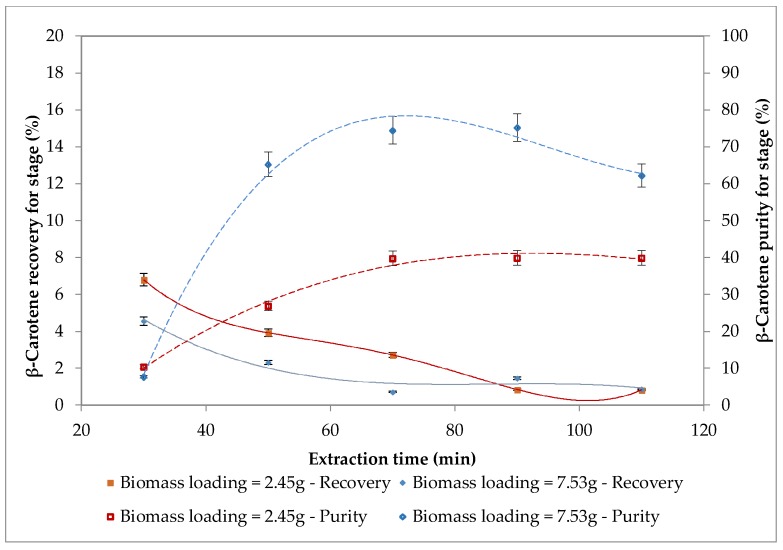
Effect of biomass loading on recovery and purity of β-carotene at 50 °C and 550 bars with a CO_2_ flow rate of 14.48 g/min.

The effect of biomass loading (2.45 and 7.53 g) on recovery and purity of β-carotene are shown in Figure 4.

It can be observed that lower biomass loading resulted in higher β-carotene recovery. A contradictory effect can be found for purity as the higher biomass loading showed maximum purity of β-carotene. With a biomass loading of 2.45 g, the highest recovery of β-carotene was equal to about 7% (first extraction cycle = 30 min, P = 550 bars, T = 50 °C, CO_2_ flow rate = 14.48 g/min), and the highest purity of β-carotene was equal to about 40% (third extraction cycle = 70 min, P = 550 bars, T = 50 °C, CO_2_ flow rate = 14.48 g/min).

With a biomass loading of 7.53 g, the highest recovery of β-carotene was close to 5% (first extraction cycle = 30 min, P = 550 bars, T = 50 °C, CO_2_ flow rate = 14.48 g/min), while the highest purity of β-carotene was equal to about 75% (fourth extraction cycle = 90 min, P = 550 bars, T = 50 °C, CO_2_ flow rate = 14.48 g/min). The lower recovery, with a biomass loading of 7.53 g, may be explained by the extraction not being fully effective because the amount of biomass inside the extraction vial was too high, this results is agreement with literature data [40,41,42].

Table 2 shows the effect of *D. salina* biomass loading on FAs recovery. Data in Table 2 show that increasing biomass loading a decrease of lipid extraction was observed. Comparing the extracted amounts with the theoretical contents, it was possible to observe that the maximum recovery of lipids, equal to 5.70 mg/g, was achieved with a biomass loading of 2.45 g, and this corresponds to a recovery of about 16% with respect to the theoretical content. The highest recovery of SFAs and MUFAs, with values of 3.3 mg/g and 1.79 mg/g, respectively, were found using a biomass loading of 2.45 g, while the highest recovery of PUFAs, equal to 0.53 mg/g, was found with a biomass loading of 7.53 g. It is worth to note that with low biomass loading (2.45 g), linoleic acid-ω-6 was extracted, while at high biomass loading (7.53 g) the presence of γ-linolenic acid-ω-6 only was detected.

### 2.4. Effect of Temperature and CO_2_ Flow Rate on β-carotene and FAs Extraction

**Figure 5:**
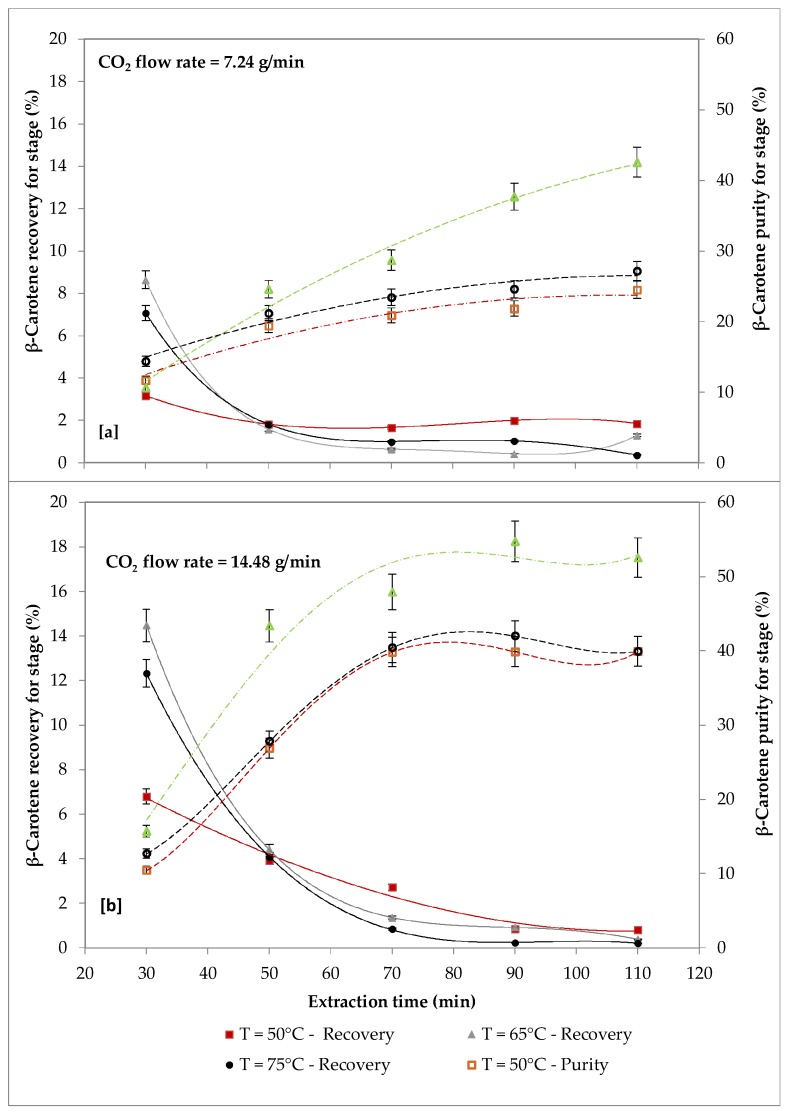
Effect of temperature (50–75 °C) on recovery and purity of β-carotene at different CO_2_ flow rates (biomass loading = 2.45 g, P = 550 bars): [a] CO_2_ flow rate = 7.24g/min; [b] CO_2_ flow rate = 14.48 g/min.

**Figure 6:**
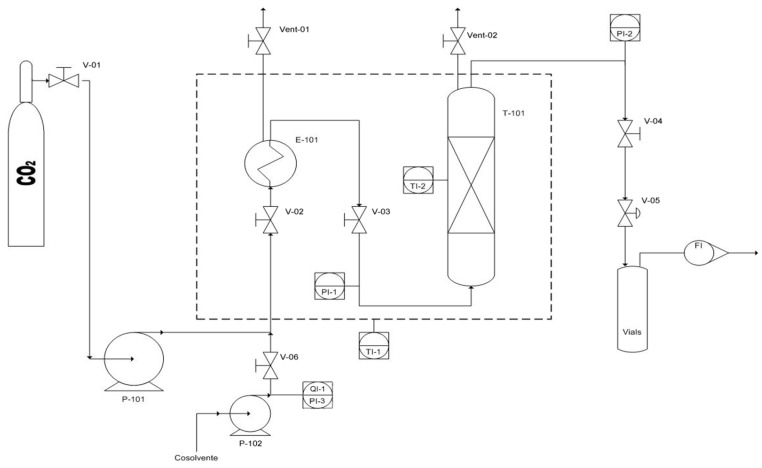
P&ID of bench scale CO_2_-SFE unit.

The effect of temperature (50 °C, 65 °C and 75 °C) with a CO_2_ flow rate of 7.24 g/min (Figure 5a) and with a CO_2_ flow rate of 14.48 g/min (Figure 5b) at 550 bars on recovery and purity of β-carotene are reported in Figure 6.

Data in Figure 5 show that with both investigated CO_2_ flow rates the recovery of β-carotene increased with temperature, reaching the highest values at 65 °C, followed by a decrease, probably because of the thermal instability of carotenoids [43]. The same trend was observed for β-carotene purity. The effect of temperature on solubility is complex, since solute and solvent characteristics should be considered [44]. In any cases, at a fixed pressure an increase in the solvent temperature causes a decrease of, solvent density, that reduces the solubility.

For all the investigated temperatures, the effect of the CO_2_ flow rate on β-carotene recovery was significant for the first extraction cycle (30 min), after which it was negligible, while β-carotene purity increased with increasing CO_2_ flow rate. In particular, about 8.6% recovery of β-carotene was achieved in first extraction cycle (30 min) with a CO_2_ flow rate of 7.24 g/min, while recovery was about 14.5% at a CO_2_ flow rate of 14.48 g/min, which was 1.7 fold higher. Similar result was reported in others study [45,46] where, during the extraction from N. oculata at 50 °C and 350 bars, a carotenoid yield increased up to19% was observed doubling CO_2_ flow rate. The effect of temperature and CO_2_ flow rate on the extraction of FAs is reported in Table 3.

With a CO_2_ flow rate of 7.24 g/min, the recovery of lipids increased, as temperature increase, achieving the highest value (8.18 mg/g) at 65 °C, followed by a decrease. A different trend was observed by combining a shorter residence time with higher CO_2_ flow rate. In fact, by using a CO_2_ flow rate of 14.48 g/min the extraction of lipid increased with increasing temperature, achieving the highest value (8.47 mg/g) at 75 °C, that is the highest temperature tested. In terms of effect of CO_2_ flow rate, experimental findings also highlighted that higher CO_2_ flow rates improved FAs recovery [46].

SFE-CO_2_ resulted in the highest extraction of SFAs (range from 1.81 to 4.93 mg/g) of biomass. Furthermore, palmitic acid was the most abundant SFA extracted in all tested conditions, comprising more than 80% of the total SFA content. Among the MUFAs, Cis-9-octadecenoic acid was the main FA extracted (32% of FAs); this finding is comparable with those in the literature. Experimental test of other researchers observed that the composition of MUFAs in microalgae can vary greatly, depending upon growth conditions [39]. With regards to PUFAs, higher concentrations of linoleic acid-ω-6 (in range of 9.17–24.22% of FAME) were obtained at the tested temperature and 550 bars with both extraction CO_2_ flow rates. 

## 3. Materials and Methods

### 3.1. Microalgal Biomass and Chemicals

The dry biomass of *D. salina* microalgae was obtained from Algalimento, Spain, with a mesh particle size of 25–50 μm and density of 0.61kg/l. The biomass was stored at −20 °C in a vacuumed plastic bag to avoid degradation and brought to room conditions before using it. The biomass of *D. salina*, characterized in terms of content of β-carotene, was measured by using the method UNI EN 12823 of 34.6 mg/g of dry biomass, and the FAs content evaluated with UNI EN ISO 12966 method, equal to 34.9 mg/g of dry biomass. Lipids, FAME (Fatty Acid Methyl Esters), SFAs, MUFAs and PUFAs extracted from *D. salina* are reported in Table 4.

CO_2_ (99.999% purity) used for supercritical fluid extraction was provided by Rivoira, Italy. The standards (β-carotene and FAs) used for the u-HPLC and GC (Santa Clara, CA United States) calibrations were analytical grade and purchased from Merck (KGaA, Darmstadt, Germany). All the solvents used were of u-HPLC grade and purchased from Merck.

### 3.2. Mechanical Pre-Treatment of Biomass

Mechanical pre-treatment of microalgal biomass was carried out by using a Retsch PM200 Retsch GmbH, Haan, Germany) planetary ball mill, with the aim to break the cell wall of *D. salina* in order to optimize β-carotene and FAs extraction yield. As reported in the literature, mechanical pre-treatment enhances the accessibility of carotenoids that bind to the cell organelles during supercritical fluids, thereby increasing extraction yield. The pre-treatment procedure was carried out by mixing 2 g of biomass with diatom earth (DE) with a range of 0.2–0.7 DE/biomass w/w as at 5 min. The effect of the pre-treatment step on β-carotene extraction was investigated by varying the grinding speed in the range 200–600 rpm and the time of pre-treatment from 2.5 to 7.5 min, in order to maximize β-carotene recovery. For the assessment of the effects of the pre-treatment, the extraction of β-carotene from *D. salina* was carried out using the ASE technique, which was considered a faster and more efficient approach for extraction. Hexane was used as an extracting solvent at 100 bars and 50 °C. Each extraction test was carried out by using 2 extraction cycles, each one of 10 min, for a total extraction time of 20 min, until complete discoloring of the biomass. It is worth highlighting that the choice of the extraction solvent was based on characteristics of the compound to be extracted; therefore, hexane was used as the extracting solvent because it is a non-polar solvent and presents the same polarity of CO_2_, which is suitable to extract a non-polar molecule like β-carotene.

### 3.3. CO_2_ Supercritical Extraction Setup

Experiment activities were carried out using a SFE-CO_2_ bench scale reactor, which was equipped with a heater, in order to achieve temperatures of up to 250 °C, and a pumping system for the compression of CO_2_ up to 680 bars. In the extraction vessel, two pressure control systems, on inlet and outlet valves (Wika Transmitter, Milano, Italy) with a precision of 0.6 mbars, were installed, while the CO_2_ flow rate was controlled using a flow meter LPN/S80 ALG 2.5 (SACOFGAS, Milan, Italy). The inlet flow rate was adjustable until 25 mL/min and the flow control was carried out on the expanded gas. Temperature was monitored by thermocouples, while inlet and outlet flow streams were controlled by micrometric valves. The experimental apparatus was also equipped with a specific line for supplying a co-solvent, using a syringe pump (Speed SFE Modifier Pump Module-PN 7170-Applied Separations, Allentown, PA) to compress the co-solvent up to 680 bars and regulate the flow rate up to 10 mL/min. The extraction vessel had a capacity of 50 mL (D = 1.35 cm, H = 35 cm), which was filled with 44 gr of glass spheres of 3 mm. In these operative conditions, biomass bulk density was about 0.119 g/mL. Furthermore, at the bottom of extraction vessel, metal frit filters, with a pore diameter of 5 μm, were used. The bench scale experimental setup is often used for preliminary tests at the end to evaluate the best operating conditions before the experimental tests on pilot scale. The Piping and Instrumentation Diagram (P&ID) of the bench scale reactor are reported in Figure 6.

Where:

- V-01/06: manual valve;
- P-101: CO_2_ pump;
- P-102: Co-solvent pump;
- E-101: Pre-Heater (Oven);
- FI: Flow meter;
- Vent-01/02: manual vent;
- T-101: Vessel for the extraction

The extraction process was carried out for 110 min and the effect of each extraction cycle (30 min) on recovery and purity of β-carotene was investigated. Different operational conditions, affecting the extraction, were investigated. In particular, temperature (T) was varied in the range 50–75° C, pressure (P) from 100 to 550 bars, CO_2_ flow rates were fixed at 7.24 or 14.48 g/min, and biomass loadings were fixed at 2.45 or 7.53 g. By using these last two biomass loadings, the bed height changed as reported in Table 5.

The effect of pressure (100–550 bar) at 50 and 65 °C, with a biomass loading of 2.45 g and CO_2_ flow rate of 14.48 g/min, was investigated. The effect of temperature was studied while operating with a biomass loading of 2.45 g and 550 bars, and a CO_2_ flow rate of 2.45 g/min and 14.48 g/min. The effect of biomass loading was explored at 50 °C and 550 bars with a CO_2_ flow rate of 14.48 g/min.

The effects of operating parameters on β-carotene extraction were expressed in terms of recovery and purity, calculated using the following equations:

(1) $$\mathrm{Recovery}\;\left(\%\right)=W_{B}/W_{T}\;\times \;100$$

(2) $$\mathrm{purity}\;\left(\%\right)=W_{B}/W_{E}\times \;100$$

where WB is the weight of β-carotene extracted (mg), as a function of the extraction time; WT is the theoretical weight β-carotene (mg); and WE is the total weight of the extract (mg).

For lipids, the effects of operating parameters were investigated on SFAs, MUFAs and PUFAs and expressed in terms of recovery, calculated as

(3) $$\mathrm{Recovery}\;\left(\mathrm{mg}/g\right)=W_{C,i}/W_{M}$$

where W_C,i_ is the weight of FAs class (mg) as a function of the extraction time; WM is the weight of microalgae on dry basis (g). For each class, the recovery was compared with respect to the theoretical content.

Each experimental condition was investigated three times and for each value the standard deviation (SD) was calculated. Finally, the extracts were stored in the dark at −80 °C to determine the total β-carotene and FAs contents using u-HPLC and GC, respectively.

### 3.4. Analytical Methods

#### 3.4.1. Analytical Methods for β-Carotene Analysis

After each extraction cycle, the sample was collected in an amber vial and subjected to basic hydrolysis in the presence of NaOH (saponification), in order to remove lipids and chlorophylls from the sample, avoiding the overlap of the spectra with the species present in the carotenoid family. In fact, literature data showed that after saponification the chromatograms of carotenoids were more comprehensible and quantifiable with respect to those obtained without saponification [47]. After saponification, β-carotene content was measured using u-HPLC Agilent 1290 Infinity II, and equipped with a diode array detector (DAD). Identification and quantification of β-carotene were carried out using an Agilent Zorbax Eclipse plus C18 column 1.8 μm column, with an isocratic mobile phase with acetonitrile and methanol (85:15 *v/v*) at a flow of 0.8 mL/min and a temperature of 30 °C for the identification of β-carotene. For the quantification of β-carotene (22040 Sigma), analytical grade standards were used, which were dissolved in chloroform with 0.1% butylated hydroxytoluene (BHT) as the antioxidant.

#### 3.4.2. Analytical Methods for FAs Analysis

The FAs analysis was carried out after the complete extraction cycle at 110 min. The extracts obtained after the SFE-CO_2_ were transesterified according to the procedure described by experimentation [32,33]. After transesterification, chromatographic analysis was carried out using a 7820A GC-FID equipped with an HP-88 100 mt × 0.25 mm × 0.2 µm column. The column was composed by high polarity bis (Cyanopropyl) siloxane stationary phase and was chosen for its high resolution of positional and geometric isomers of FAME. The temperature of the injector and detector was controlled at 250 °C and that of the oven from 150 °C to 240 °C with a ramp of 4 °C/min. Nitrogen (purity > 99.999%) was used as a gas carrier with a spatial velocity of 30 cm/s split ratio of 1:100. An internal analytical standard, the heneicosanoic acid (C:21) purchased form SIGMA-Aldrich (H5149) (Sigma-Aldrich Ltd., St. Louis, MO, USA), was used to quantify fatty acid methyl esters and a mixture of 37 fatty acid ethyl esters (C4–C24) (Supelco FAME 37, CRM47885, Sigma-Aldrich Ltd., St. Louis, MO, USA) was used for the qualitative analysis.

## 4. Conclusions

In this work, the effect of mechanical pre-treatment and operating parameters on the extraction of β-carotene and FAs from *D. salina* using SFE-CO_2_ was investigated. Moreover, the extraction capacity of SFE-CO_2_ on FA species, distinguished as SFAs, MUFAs and PUFAs, was also evaluated. Results highlighted that the mechanical pre-treatment of microalgae was required in order to enhance β-carotene extraction.

The extraction pressure showed an opposite effect of recovery and purity of β-carotene, in particular increasing the extraction pressure, the purity of β-carotene increased (with the highest value at 550 bars), while the recovery of β-carotene achieved the highest value at 400 bar, after which it decreased. By increasing the biomass loading, a decrease of β-carotene recovery was observed. The recovery of β-carotene increased with temperature until 65 °C, after which it decreased. The same trend was observed for the purity of β-carotene. β-carotene recovery was affected by the CO_2_ flow rate for the first extraction cycle (30 min), after which the effect of the CO_2_ flow rate was negligible, while β-carotene purity increased with an increasing CO_2_ flow rate.

The highest lipid recovery was obtained at an extraction pressure of 400 bars, with CO2 flow rate of 7.24 g/min and an extraction temperature of 65 °C. Overall the results show that the operating extractive conditions have to be accurately chosen in order to obtain the highest values of purity and recovery.

## Figures and Tables

**Table 1:** Effect of pressure (100–550 bars) on recovery of FAs at 50 °C and 65 °C (biomass loading = 2.45 g, CO_2_ flow rate = 14.48 g/min, extraction time = 110 min).

Class of Fatty Acids	Operative Temperature (°C)					
	50			65		
	Operative Pressure (bar)					
	100	400	550	100	400	550
Lipids (mg/g)	1.65 ± 0.07	6.37 ± 0.26	5.70 ± 0.23	1.24 ± 0.05	7.91 ± 0.32	0.88 ± 0.04
% of FAME	94.06 ± 4.33	91.73 ± 4.22	98.66 ± 4.54	89.38 ± 4.11	95.88 ± 4.41	96.21 ± 4.43
FAME (mg/g)	1.55 ± 0.05	5.84 ± 0.19	5.62 ± 0.18	1.11 ± 0.04	7.58 ± 0.24	0.84 ± 0.03
SFAs (mg/g)	0.79 ± 0.02	3.00 ± 0.07	3.30 ± 0.08	1.00 ± 0.02	4.12 ± 0.10	0.55 ± 0.01
MUFAs (mg/g)	0.62 ± 0.01	2.17 ± 0.09	1.79 ± 0.07	0.11 ± 0.00	2.69 ± 0.11	0.23 ± 0.01
PUFAs (mg/g)	0.14 ± 00	0.67 ± 0.02	0.53 ± 0.02	<Ldl	0.78 ± 0.03	0.06 ± 0.00
**SFAs**						
Palmitic acid (% of FAME)	44.15 ± 2.21	43.54 ± 2.18	50.12 ± 2.51	85.83 ± 4.29	45.29 ± 2.26	65.58 ± 3.28
Palmitic acid (mg/g)	0.68 ± 0.03	2.54 ± 0.11	2.81 ± 0.12	0.95 ± 0.04	3.43 ± 0.14	0.55 ± 0.02
Stearic acid (% of FAME)	6.89 ± 0.28	7.83 ± 0.32	8.50 ± 0.35	4.47 ± 0.18	8.96 ± 0.37	<Ldl
Stearic acid (mg/g)	0.11 ± 00	0.46 ± 0.02	0.49 ± 0.02	0.05 ± 0.00	0.69 ± 0.03	<Ldl
**MUFAs**						
Cis-9-Octadecenoic acid (% of FAME)	39.80 ± 1.79	37.14 ± 1.67	31.89 ± 1.44	9.70 ± 0.44	35.44 ± 1.59	27.41 ± 1.23
Cis-9-Octadecenoic acid (mg/g)	0.62 ± 0.01	2.17 ± 0.03	1.79 ± 0.03	0.11 ± 0.00	2.69 ± 0.12	0.23 ± 0.00
**PUFAs**						
Linoleic acid-ω-6 (% of FAME)	9.16 ± 2.5	11.49 ± 0.29	9.49 ± 0.24	<Ldl	10.31 ± 0.26	7.00 ± 0.18
Linoleic acid-ω-6 (mg/g)	0.14 ± 1.5	0.67 ± 0.01	0.53 ± 0.01	<Ldl	0.78 ± 0.04	0.06 ± 0.00
γ-Linolenic acid-ω-6 (% of FAME)	<Ldl	<Ldl	<Ldl	<Ldl	<Ldl	<Ldl
γ-Linolenic acid-ω-6 (mg/g)	<Ldl	<Ldl	<Ldl	<Ldl	<Ldl	<Ldl

Note: FAME = fatty acid methyl ester; SFAs = saturated fatty acids; MUFAs = monounsaturated fatty acids; PUFAs = polyunsaturated fatty acids; <Ldl = lower than the detection limit.

**Table 2:** Effect of biomass loading (2.45 g, 7.53 g) on recovery of FAs (P = 550 bars, T = 50 °C, CO_2_ flow rate = 14.48 g/min, extraction time = 110 min).

Class of Fatty Acids	Biomass Loading (g)	
	2.45	7.53
Lipids (mg/g)	5.70 ± 0.23	3.73 ± 0.15
% of FAME	98.66 ± 4.54	89.61 ± 1.97
FAME (mg/g)	5.62 ± 0.18	3.34 ± 0.13
SFAs (mg/g)	3.30 ± 0.08	2.44 ± 0.10
MUFAs (mg/g)	1.79 ± 0.07	0.18 ± 0.01
PUFAs (mg/g)	0.53 ± 0.02	0.72 ± 0.03
**SFAs**		
Palmitic acid (% of FAME)	50.12 ± 2.51	73.20 ± 2.64
Palmitic acid (mg/g)	2.81 ± 0.12	2.44 ± 0.10
Stearic acid(% of FAME)	8.50 ± 0.35	<Ldl
Stearic acid (mg/g)	0.49 ± 0.02	<Ldl
**MUFAs**		
Cis-9-Octadecenoic acid (% of FAME)	31.89 ± 1.44	5.34 ± 0.19
Cis-9-Octadecenoic acid (mg/g)	1.79 ± 0.03	0.18 ± 0.01
**PUFAs**		
Linoleic acid-ω-6 (% of FAME)	9.49 ± 0.24	<Ldl
Linoleic acid-ω-6 (mg/g)	0.53 ± 0.01	<Ldl
γ-Linolenic acid-ω-6 (% of FAME)	<Ldl	21.46 ± 0.54
γ-Linolenic acid-ω-6 (mg/g)	5.70 ± 0.23	0.72 ± 0.03

Note: FAME = fatty acid methyl ester; SFAs = saturated fatty acids; MUFAs = monounsaturated fatty acids; PUFAs = polyunsaturated fatty acids; <Ldl = lower than the detection limit.

**Table 3:** Effect of different operative temperatures at 550 bars with CO_2_ flow rates of 7.24 and 14.48 g/min for FAs recovery (extraction time = 110 min).

Class of Fatty Acids	Operative CO_2_ Flow Rate at 550 bars					
	7.24 g/min			14.48 g/min		
	Operative Temperature (°C)					
	50	65	75	50	65	75
Lipids (mg/g)	3.05 ± 0.15	8.18 ± 0.34	4.45 ± 0.14	5.70 ± 0.23	7.75 ± 0.24	8.47 ± 0.34
% of FAME	96.59 ± 2.51	95.65 ± 1.34	94.06 ± 1.50	98.66 ± 4.54	97.07 ± 0.87	95.96 ± 1.54
FAME (mg/g)	2.94 ±0.12	7.82 ± 0.38	4.18 ± 0.16	5.62 ± 0.18	7.52 ± 0.32	8.12 ± 0.26
SFAs (mg/g)	1.81 ± 0.08	4.62 ± 0.22	2.54 ± 0.11	3.30 ± 0.08	4.34 ± 0.19	4.93 ± 0.17
MUFAs (mg/g)	0.42 ± 0.02	2.22 ± 0.10	1.26 ± 0.06	1.79 ± 0.07	2.32 ± 0.08	2.46 ± 0.11
PUFAs (mg/g)	0.71 ± 0.03	0.99 ± 0.04	0.38 ± 0.02	0.53 ± 0.02	0.87 ± 0.03	0.73 ± 0.03
**SFAs**						
Palmitic acid (% of FAME)	56.48 ± 1.19	47.53 ± 0.52	54.42 ± 0.98	50.12 ± 2.51	50.06 ± 0.75	53.71 ± 0.86
Palmitic acid (mg/g)	1.66 ± 0.08	3.72 ± 0.17	2.27 ± 0.11	2.81 ± 0.12	3.76 ± 0.18	4.36 ± 0.18
Stearic acid (% of FAME)	5.02 ± 0.24	11.50 ± 0.48	6.46 ± 0.23	8.50 ± 0.35	7.71 ± 0.78	7.02 ± 0.34
Stearic acid (mg/g)	0.15 ± 0.01	0.90 ± 0.04	0.27 ± 0.01	0.49 ± 0.02	0.58 ± 0.03	0.57 ± 0.03
**MUFAs**						
Cis-9-Octadecenoic acid (% of FAME)	14.28 ± 0.50	28.38 ± 0.54	30.20 ± 1.06	31.89 ± 1.44	30.81 ± 0.77	30.30 ± 1.36
Cis-9-Octadecenoic acid (mg/g)	0.42 ± 0.02	2.22 ± 0.08	1.26 ± 0.05	1.79 ± 0.03	2.32 ± 0.10	2.46 ± 0.04
**PUFAs**						
Linoleic acid-ω-6 (% of FAME)	24.22 ± 0.36	12.59 ± 0.23	9.17 ± 0.23	9.49 ± 0.24	10.77 ± 0.48	8.98 ± 0.22
Linoleic acid-ω-6 (mg/g)	0.71 ± 0.03	0.99 ± 0.04	0.38 ± 0.02	0.53 ± 0.01	0.81 ± 0.04	0.73 ± 0.04
γ-Linolenic acid-ω-6 (% of FAME)	<Ldl	<Ldl	<Ldl	<Ldl	0.76 ± 0.04	<Ldl
γ-Linolenic acid-ω-6 (mg/g)	<Ldl	<Ldl	<Ldl	<Ldl	0.06 ± 0.00	<Ldl

Note: FAME = fatty acid methyl ester; SFAs = saturated fatty acids; MUFA = monounsaturated fatty acids; PUFA = polyunsaturated fatty acids; <Ldl = lower than the detection limit.

**Table 4:** Characterization of fatty acids from *Dunaliella salina* biomass.

Class of Fatty Acids	Theoretical Content
Lipids (mg/g)	34.87 ± 1.05
% of FAME	90.51 ± 3.62
FAME (mg/g)	31.56 ± 1.10
SFAs (mg/g)	15.33 ± 0.69
MUFAs (mg/g)	05.68 ± 0.28
PUFAs (mg/g)	10.56 ± 0.31
SFAs	
Palmitic acid (% of FAME)	30.57 ± 1.25
Palmitic acid (mg/g)	09.65 ± 0.44
Stearic acid (% of FAME)	17.99 ± 0.58
Stearic acid (mg/g)	05.68 ± 0.14
MUFAs	
Cis-9-Octadecenoic acid (% of FAME)	17.98 ± 0.52
Cis-9-Octadecenoic acid (mg/g)	05.68 ± 0.12
PUFAs	
Linoleic acid-ω-6 (% of FAME)	16.47 ± 0.68
Linoleic acid-ω-6 (mg/g)	05.2 ± 0.22
γ-Linolenic acid-ω-6 (% of FAME)	16.99 ± 0.70
γ-Linolenic acid-ω-6 (mg/g)	05.36 ± 0.17

**Table 5:** Characteristics of bed after mixing with glass spheres and diatom earth (DE).

Biomass Loading (g)	DE (g)	Glass Spheres (g)	Bulk Density (g/mL)	Bed Height (cm)	Porosity [-]
2.45	0.98	44.0	0.087	19.7	0.46
7.53	3.01	44.0	0.223	23.6	0.44
